# From pox to protection: understanding Monkeypox pathophysiology and immune resilience

**DOI:** 10.1186/s41182-025-00708-6

**Published:** 2025-02-26

**Authors:** Alagammai Ganesan, Thirumalai Arunagiri, Suganandhini Mani, Vamsi Ravi Kumaran, Kanaka Parvathi Kannaiah, Hemanth Kumar Chanduluru

**Affiliations:** SRM College of Pharmacy, SRM Institute of Science and Technology, Kattankulathur, Chennai, Tamil Nadu 603203 India

**Keywords:** Monkeypox, Orthopoxvirus, Pathophysiology, Immunity, Immune evasion, Immunotherapeutics

## Abstract

The Monkeypox virus (MPXV), which causes Monkeypox (Mpox) is an invasive ailment with global implications. MPXV, categorized within the Orthopoxvirus genus, exhibits diverse clades with varying fatality rates. Initially discovered in monkeys and later in humans, the disease predominantly affects regions across West and Central Africa. Clinical manifestations encompass a spectrum from mild flu-like symptoms to severe eruptions. This article aims to give the scientific community a comprehensive overview of Mpox Pathophysiology and delve into the intricate landscape of host immune responses against MPXV infection. It offers crucial insights into the virus's Pathophysiology, spanning its entry, replication, dissemination, and elicited host responses. The immune reaction to Mpox involves innate immunity, B-cell immunity, and T-cell immunity. Moreover, this review underscores the immunological response and resistance mechanisms against MPXV. It also highlights imperative research areas warranting prioritization to devise more efficacious treatments for controlling viral propagation within healthcare systems. In addition, it gives us a look into possible futures that could help the progress of immunotherapies and cutting-edge biotechnological solutions for protecting against MPXV.

## Introduction

The emergence of viral threats is an enormous challenge for public health systems globally in the ever-changing environment of infectious diseases. Among these challenges has been the reemergence of Monkeypox (Mpox) and orthopoxvirus infections with potential cross-species transmission. Mpox was named after a smallpox-like illness in macaques that occurred in a Danish research institution in 1958. The first human Mpox infection was discovered in 1970 in the country of the Democratic Republic of Congo (DRC) [1], previously known as Zaire. As a result, Central and Western Africa, with their dense tropical rainforests and abundant virus-carrying organisms, emerged as primary regions for Mpox outbreaks. In the meantime, many novel Mpox species have been discovered, mostly in African countries [2, 3]. Globally, Monkeypox virus (MPXV) cases have grown substantially since May 2022 [4]. On July 23, 2022, the World Health Organization (WHO) designated the Mpox outbreak as a worldwide health emergency. Mpox is triggered by the MPXV, a virus from the Orthopoxvirus genus of the Poxviridae group. This virus is linked to other orthopoxvirus species, notably cowpox, vaccinia virus (VACV) (used in the smallpox vaccine), and variola (the virus that causes smallpox, which has been eradicated) [5].

The evolutionary MPXV can be classified into two basic clades: Central African (also referred to as Congo Basin or clade I) and West African (Clade II). Additionally, Clade II is divided into two groups: IIa and IIb. The IIb is now spreading internationally through human transmission, although Clade IIa is indigenous to West Africa and possesses a low fatality rate. The sequence similarity among West African MPXV clades can range from ~ 95% to > 99%, depending on their origin. Assuming an average fatality rate of 10.6% compared to 3.6% over the West African clade, the Central African clade is expected to be more virulent [6, 7]. The most recent epidemic is being investigated to establish the presence of genetic mutations in the MPXV genome [8]. With outbreak clusters growing every day, the current MPXV outbreak is spreading at an unprecedented rate [9]. According to WHO data, 102,977 confirmed instances of Mpox triggered by MPXV clades I and II, involving 219 fatalities, have been recorded across 121 countries between the beginning of Mpox surveillance in 2022 and July 31, 2024. Singapore recorded its first imported human Mpox infection connected to the 2022 global outbreak, following a prior case imported from Nigeria in 2019. Numerous cases, both local and imported, were subsequently confirmed [10]. According to a landmark study conducted in the DRC in 1988, those who obtained the smallpox vaccine experienced an 85% lower chance of contracting Mpox than those who did not get the vaccination, as per a state-wide program that started 12 years prior to data collection [9]. Interestingly, one feature of the most recent outbreaks is the unusually high rate of infection among men who have sex with men (MSM) [11, 12].

The scientific community has been prompted by the recent increase of cases in 2022 to investigate the origins, pathophysiology, etiology, immunity, and management strategies of Mpox outbreaks. Furthermore, the scientific community is under increasing pressure to increase knowledge and investigate the threat, since the WHO deemed the Mpox pandemic an international health crisis. This compilation attempts to give the scientific community an overview of the pathology associated with Mpox as well as an overview of the host immune responses that must be taken into account in the battle against Mpox infection. Additionally, this article provides insights into the influence of circadian rhythm on vaccine efficacy, immunity, and immune response, emphasizing the role of biological clocks in optimizing immunological outcomes. Furthermore, this work will call attention to the research gaps that need to be filled to make advanced biotechnological conclusions about the virology of Mpox and to develop better treatments for managing viral loads in healthcare systems in the future [13].

## Etiology

In western and central Africa, exposure to rats, rabbits, squirrels, monkeys, porcupines, and gazelles have been linked to outbreaks. Direct contact with diseased animals during their capture, killing, and/or preparation for food, in addition to ingestion, may expose people in remote tropical rainforests to disease. Consuming such so-called "bush meat" is extremely harmful, since the flesh is commonly undercooked. Because of the range of animals ingested by the local people, conclusions on the relative hazards of different meat sources are doubtful [14].

## Mpox pathology

The MPXV infection often manifests as fever (in the range of 38.5 degrees Celsius and 40.5 degrees Celsius), a headache, and myalgia, and it takes 5–21 days to incubate [15]. One characteristic that sets MPXV infection apart is lymphadenopathy, or enlargement of the inguinal, cervical, or maxillary lymph nodes [16]. As soon as the fever begins, rashes spread throughout the body. Lesions in the oral cavity may lead to problems while eating and drinking later in the course of the illness [2]. However, subsequent outbreaks have reported some surprising clinical results. In MSM individuals, they include the occurrence of anal ulcers that migrate to other body areas [17].

Lesions often progress via four stages: macular, papular, vesicular, and pustular, before peeling off as scabs [18]. Patients are often considered non-contagious once the lesions become crusted over. However, it has been discovered that scabs retain significant levels of MPXV DNA long after lesions have been removed, suggesting the presence of contagious viral material. It is interesting to note that live Variola virus was found in smallpox sufferers' scabs [13]. During pregnancy, MPXV can be transferred directly from the pregnant woman to the fetus. There is little possibility of carrying a healthy child. In a same way, studies on toddlers showed a deadly strain of Mpox that had a higher death rate than that of older adults. The poorer immune systems of children may be directly linked to this disparity [19].

### Two different phases comprise the MPXV infections

The prodromal phase, which lasts 0–3 days and starts 4–17 days after virus exposure, is marked by enlarged lymph nodes, a high temperature (fever), migraines, chills, exhaustion, muscle aches, back pain, and the itching phase [11], which lasts for 7–21 days and starts 3 days after the prodromal phase [20]. The typical manifestations of Mpox are a painful or itchy maculopapular rash that develops into a vesiculopustular lesion [12].

## Pathophysiology of MPXV

Close interaction involving animals and people or human-to-human transmission is the first step in the pathogenesis and physiology of the MPXV. MPXV infects its host by taking use of multiple entry points, including the subcutaneous portals, oropharynx, and nasopharynx [21]. After entering the body, the virus multiplies there before spreading to neighboring lymph nodes. MPXV extends its reach to other organ systems as the infection progresses after an initial phase of viral invasion [22]. Despite its DNA nature, MPXV carries out its entire life cycle within the host cell's cytoplasm. This intricate process involves numerous proteins essential to viral DNA replication, transcription, and virion packaging [23]. The virus employs diverse mechanisms to enter host cells, including fusion and macropinocytosis [20].

MPXV exhibits two different infectious virions: extracellular enveloped virus (EVV) and intracellular mature virus (IMV) [17]. From the original site of infection, the virus travels to drainage lymph nodes that have immediate communication with lymphatic channels and antigen-presenting cells. MPXV targets spleen and liver, resulting in a more prominent second wave of viremia after an early period of viral replication inside lymph nodes produces a modest primary viremia. The virus can spread to farther-off organs like the lungs, kidneys, intestines, and skin due to this elevated viremia. Notably, in models of respiratory-acquired Clade 1 MPXV infections in non-human primates, the respiratory tract plays a critical role [25].

**Fig. 1:**
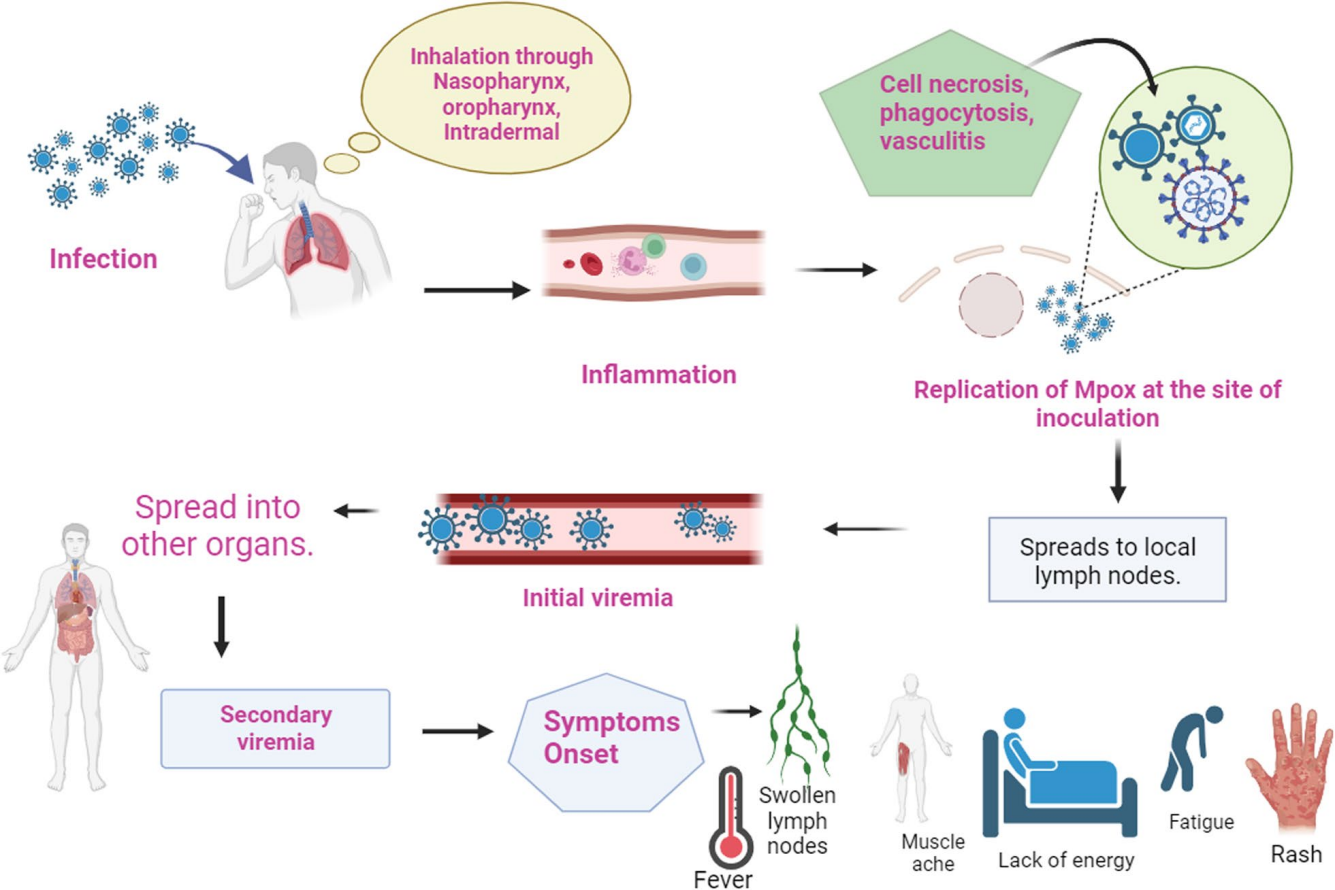
The infectious viral disease caused by MPXV spreads by direct contact between humans and animals or from person to person via several of the entrance sites, it multiplies, spreads to lymph nodes, and causes primary or initial viremia in the host. The virus has two distinct infectious virions: IMV and EEV. It targets larger organs like the liver and spleen. For Mpox, the incubation period is usually up to 21 days. Fever, headaches, pains in the muscles, enlarged lymph nodes, and a rash that takes the form of pustular lesions are some of the clinical symptoms

Subcutaneous inoculation models in primates have demonstrated that clade 2 MPXV infection mostly causes moderate, localized illness, because viral replication takes place in the skin and lymphatic system [26]. Remarkably, clade 1 MPXV can affect the gastrointestinal, respiratory, and genitourinary systems even after skin inoculation [27]. During the 2022 outbreak, individuals experienced localized oral and anogenital lesions as a result of viral transmission through sexual contact. While widespread distributed skin lesions were extremely uncommon, some people had a few distant lesions on their face, limbs, and trunk [28]. This intricate interplay between viral entry, replication, dissemination, and host responses contributes to the complex pathophysiology of MPXV infection as depicted in Fig. 1, underscoring the importance of understanding these dynamics for developing effective diagnostic, treatment, and prevention strategies.

## Immunity to MPXV

Although MPXV was discovered some decades ago, little is known about human immunity to it. VACV and similar orthopoxviruses are frequently used as models in studies on MPXV's interactions with the host system of immunity. This review explores the immune evasion tactics used by MPXV during active infection as well as possible host immunity mechanisms against MPXV. Understanding how people react to MPXV is still challenging due to a lack of information, and hence, studies that combine VACV and orthopoxviruses are commonly used to gain understanding [4]**.**

### MPXV-induced immune responses

In addition to being the initial line of resistance against viral infections, innate immune cells can also be targets for particular viruses. Studies conducted within in vitro and in vivo have shown that monocytes in particular are the main targets of poxviruses [22, 23]. One reliable sign of virus lethality is the existence of poxvirus antibodies in neutrophils and monocytes [24]. Furthermore, it has been discovered that the human main M2-like macrophages aid in the reproduction and spread of VACV [29].

The natural killer (NK) cells and monocytes have an impact on adaptive immune responses; NK cells are essential elements of innate immunity [26]. NK cells separated from lymph nodes and blood, however, can show less degranulation and less release of tumor necrosis factor (TNF) and interferon-gamma (IFN-γ) [27]. Clarifying the functions of different innate immune cells, such as neutrophils, NK cells, dendritic cells, monocytes/macrophages, and innate lymphoid cells, is essential for finding possible biomarkers and learning more about the prognosis of the disease, even though these functions are not entirely understood in MPXV-infected humans [4]. Tumor necrosis factor alpha (TNF-α), IFN-γ, IL-2, and IL-12 are significantly reduced in human MPXV infection, but ILs, C–C motif chemokine ligand 2 (CCL2), and CCL5 are elevated. IgM and IgG antibodies are produced by MPXV infection, residual IgG-memory B cells remain for a long time, and activated effector CD4 + and CD8 + T cells proliferate rapidly thereafter gradually declining. By suppressing T-cell receptor-mediated activation of T cells, MPXV also impedes antiviral CD8 + and CD4 + T-cell responses, which are adaptive immune response [30].

### B cells and defense against antibodies

During the worldwide smallpox eradication effort with the live VACV vaccine, the critical function of B lymphocytes and immunoglobulins in combating poxviruses was highlighted. Those who were in close contact with those suffering from smallpox were effectively protected from infection due to the efficient usage of vaccinia immune globulin (VIG), thereby is produced from vaccine recipients' serum. VACV-specific B-cell responses protected rhesus macaques against deadly MPXV infections. Epidemiological studies have also demonstrated that VACV immunization provides protection against a variety of poxviruses, including MPXV. The memory B cells and persistent antibody production against VACV created during vaccination have demonstrated exceptional robustness and durability, with protecting qualities observed for over 50 years in some cases [31]. These findings highlight the enduring influence of B-cell-mediated immunity and immunoglobulin responses in conferring broad protection against poxviruses [32].

Nonetheless, the cross-protective immunity against smallpox appears to wane over time, as approximately half of the vaccinated individuals more than two decades after vaccination exhibited suppressed neutralizing antibody titers [31]. Immunoglobulins induced by the cross-reactive VACV recognition of human vaccine recipients recognized fourteen MPXV proteins. The composition of the anti-MPXV response, characterized by the prevalence of IgM antibodies during primary immune reactions and IgG antibodies during secondary responses, could offer valuable insights into existing immunity and protective mechanisms. IgM responses might also indicate disease severity, emphasizing the importance of extensively profiling antibody responses within diverse MPXV patient cohort. B cells, despite being less directly competitive with poxviruses than T cells, play a critical role as specialized antigen presenting cells by upregulating costimulatory ligands such as CD28 and Inducible Co-stimulator, which are essential for the activation and proliferation of poxvirus-specific CD4 + and CD8 + T cells. These interactions also influence the activation of B cells and contribute to the development of T follicular helper cells, highlighting their interconnected roles in immune regulation. Furthermore, B cells produce a diverse array of neutralizing antibodies (NAbs) against MPXV infection, targeting various viral components, including IMV and EEV. However, EEVs demonstrate significant resistance to complement mediated neutralization, reducing the efficacy of NAbs in the absence of complement enhancement. Advances in immunoinformatics have facilitated the prediction of EEV membrane components, thereby aiding in the rational design and development of multi-epitope vaccines against MPXV [33, 34].

### T-cell immunity

Follicular assistance cells (CD4 + T cells) play an important role in memory B-cell development and antibody production. After a VACV vaccination, these cells can continue to produce TNF and IFN-γ for a maximum of 50 years. It is fascinating to consider that CD4 + T cells are crucial for producing protective responses against deadly MPXV outbreaks in rhesus macaques inoculated with VACV [35]. Furthermore, CD8 + T cells have been examined in a mice model of VACV infection to remove pathogen-infected leukocytes and decrease virus growth, suggesting that both CD4 + and CD8 + T cells play a direct role in antiviral activities. It is essential to remember that smallpox immunization does not ensure robust T-cell-mediated protection to MPXV. The exact link among human MPXV infection severity as well as CD4 + and CD8 + T-cell activation remains uncertain [4]. T-cell-mediated immunity constitutes a pivotal aspect of the adaptive immune system, primarily regulated through major histocompatibility complex (MHC) molecules that drive the activation and differentiation of CD4 + and CD8 + T cells. Although MPXV does not directly target MHC molecules, it employs alternative mechanisms to evade T-cell responses. The MPXV B22 protein impairs T-cell-mediated control of viral dissemination, while its M2 protein inhibits costimulatory signals by binding to B7 ligands, thereby suppressing CD28-mediated activation more effectively than CTLA4. This dual suppression significantly diminishes the activation of CD4 + and CD8 + T cells, disrupting germinal center formation and B-cell maturation key processes essential for a robust adaptive immune response. Additionally, the M2 protein enhances PD-L1 stimulation, further exacerbating T-cell exhaustion and immune dysfunction. Notably, the modified vaccinia Ankara strain (MVA-BN) does not produce functional M2 protein, resulting in enhanced CD4 + and CD8 + T-cell responses compared to MPXV and wild-type VACV. These findings underscore the potential of genetic modifications to eliminate inhibitory genes from MVA, further improving its efficacy as a vaccine and promoting robust T-cell-mediated immunity against MPXV [36].

## Immune evasion of MPXV

**Fig. 2:**
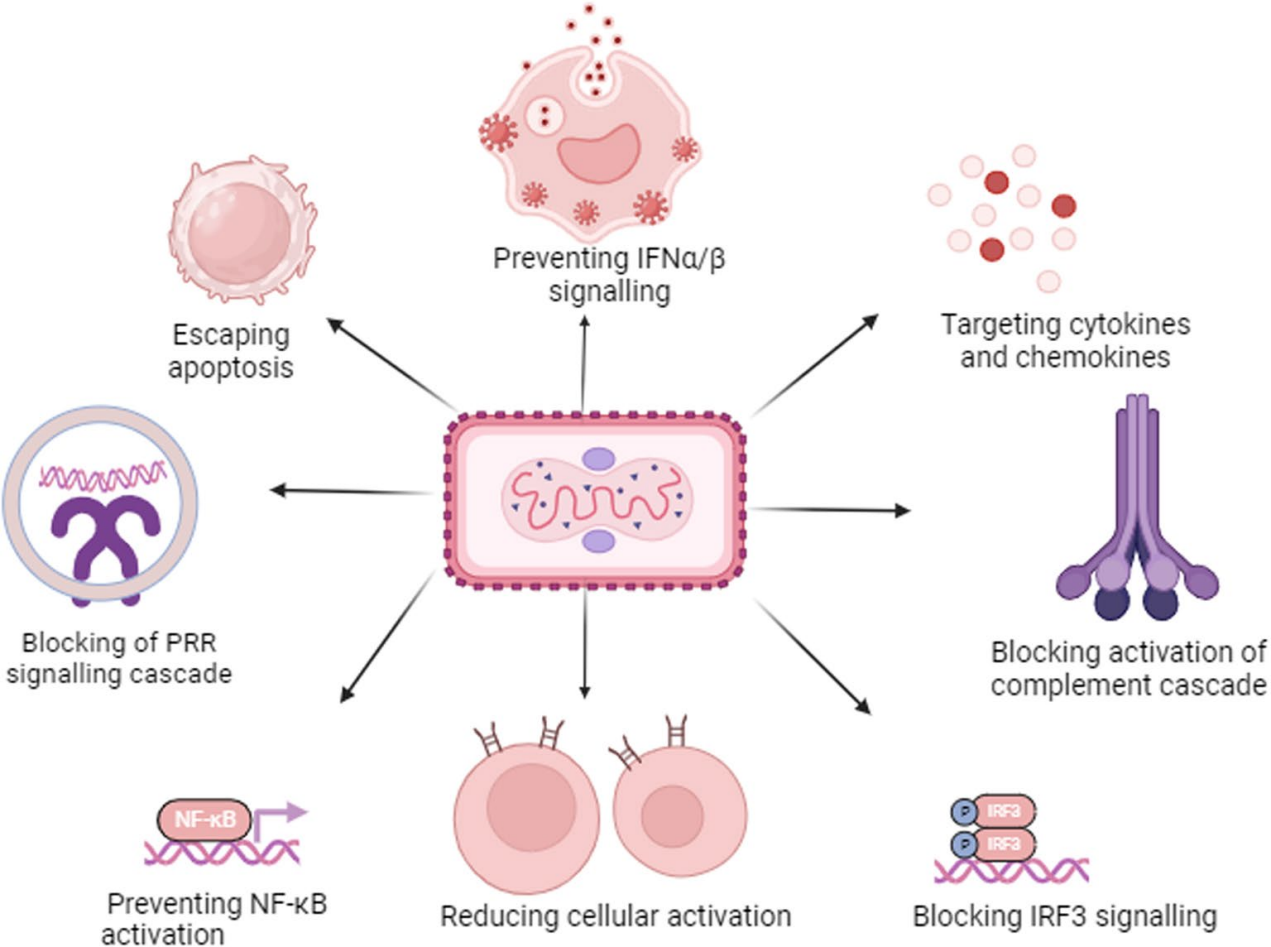
Many of the MPXV's proteins interact with infectious agent receptor signaling cascades, controlling the expression of pro-inflammatory genes with the value as nuclear factor kappa B cells (NF-B) and interferon-regulated factor 3 (IRF3). This allows the virus to avoid protecting individuals. MPXV reduces interferon signaling by blocking IFN/binding, IFN/production, and PKR-mediated pathways. It produces antibodies that target inflammatory molecules such as IFN gamma, TNF, IL-18, IL-6, and IL-1 beta. MPXV may avoid infected cells from dying by producing proteins that inhibit apoptotic pathways. It also reduces T-cell and natural killer activation

Orthopoxviruses have evolved a set of genes that encode proteins that alter hosting cell signaling pathways important in viral identification, apoptosis, and immunological control. Figure 2 vividly displays the whole picture of evasion of immunity.

### Cellular signaling prevention

**Fig. 3:**
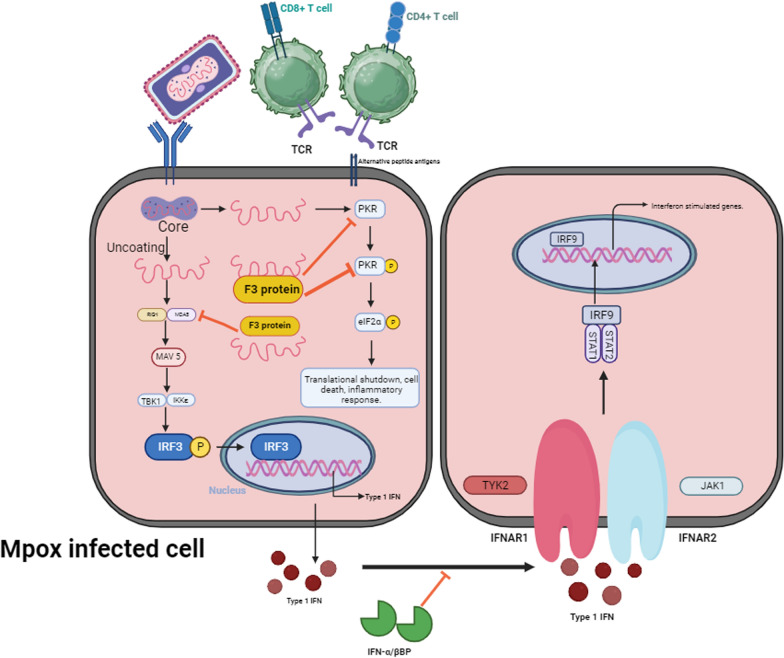
Pattern recognition receptor (PRRs) in the host cell population detect the MPXV infection by triggering the synthesis of type I interferons (IFNs). These IFNs induce an antiviral response by activating gene expression via the JAK–STAT signaling mechanism. The MPXV-encoded F3 protein inhibits IFN signaling and circumvents the immune response. It also blocks IFN from binding to cellular receptors, allowing antiviral CD4 + and CD8 + responses from T cells to be avoided. STAT (Signal Transducer and Promoter of Transcription), JAK1 (Janus Kinase 1), and IKKε (IкB Kinase Epsilon) are all acronyms. P = phosphorylation, TANK-Binding Kinase 1 (TBK1), Interferon Regulatory Factor (IRF). TYK2 stands for Tyrosine Kinase 2. The acronyms IFNAR (Interferon-Alpha/Beta Receptor), MAVS (Mitochondrial Antiviral Signaling), and eIF2α (Eukaryotic Translation Initiation Factor 2)

MPXV's immune evasion is likely a critical factor in its propagation and mutation. The deep genetic similarity between Orthopoxvirus orthologues and MPXV suggests that the virus employs analogous tactics to escape host immune responses [37]. Interferons (IFNs), pivotal cytokines that inhibit viral replication, form a critical part of the innate immune response. MPXV produces proteins that hinder the activation of IFN pathways, weakening the host's ability to alert neighbouring cells about viral infection and thus dampening early immune responses [38]. MPXV circumvents the host's antiviral innate immunity by suppressing type I IFN responses, as seen in Fig. 3 [39]. Proinflammatory chemicals involved in antiviral responses are produced when Toll-like receptors (TLRs) detect damage-associated molecular patterns (DAMPs), such as viral double-stranded RNA (dsRNA), activating the adaptive immune system. The MPXV orthologue A47 resembles the VACV protein A52R, which can inhibit both TLR3 and TLR4 signaling pathways, while the VACV A46 protein specifically disrupts TLR4 signaling. Although MPXV produces limited dsRNA intermediates, whether TLR3 can recognize them remains unclear. Additionally, dsRNA activates protein kinase R (PKR), which phosphorylates eIF2α, halting the translation of cellular and viral mRNA. VACV proteins E3 and K3 inhibit PKR, preventing an antiviral response. Furthermore, IFNs play a critical role in viral replication inhibition. The MPXV B16 protein acts as a secreted inhibitor of the type I IFN-induced signaling cascade. The cGAS/STING DNA-sensing receptor pathway can also activate NFκB and IFN pathways. MPXV evades these responses by targeting the antiviral cytokine TNFα and other immunomodulatory molecules. The cytokine response-modifying protein B (CrmB) encoded by MPXV serves as a decoy receptor for TNFα, disrupting immune system activation [30].

### Apoptosis regulation

Immune evasion also involves manipulating apoptosis. Orthopoxviruses use BCL-2-like proteins to disrupt the control exerted by BCL-2 over the intrinsic apoptotic pathway. Furthermore, various orthopoxvirus-encoded serine protease inhibitors (serpins) modulate this cellular process [40].

### Immune mediator antagonism

Orthopoxviruses use a variety of tactics to overcome the host immune system's defenses, such as secreting proteins that disrupt immune mediators such as chemokines, IL-1β, IFN-γ, and the complement system [41]. MPXV strains from Central Africa have Mpox inhibitors of complement enzymes (MOPICE), whereas strains from West Africa lack complement-modulating proteins [42]. Significantly, MOPICE inhibits complement activation, but its deletion does not affect virulence [41]. In mice infected with MPXV, early activation of the complement system is crucial for viral control. However, MPXV modifies this response by blocking the complement pathway using MOPICE, which suppresses the adaptive immune system and promotes viral replication in rhesus macaques. The absence of the D14L gene in Clade II renders the virus more susceptible to complement attack. Additionally, MPXV disrupts adaptive immunity by preventing MHC class II-restricted antigen presentation and T-cell receptor-mediated T-cell activation [43].

### MHC manipulation and suppression

Orthopoxviruses use techniques to limit the function of their cells. One such strategy is to create the orthopoxviruses major histocompatibility complex, also known as MHC class I-like amino acids, which is generated by the N3R gene and inhibits T-cell cytotoxicity. This approach lowers MHC class I synthesis and NK cell lysis, which impairs T-cell identification. Additionally, MPXV may effectively reduce T-cell-mediated immune system functions by causing T-cell unresponsiveness via an MHC-independent mechanism [4, 35].The MPXV protein B10 interferes with peptide loading and MHC-I trafficking in the endoplasmic reticulum, preventing cytotoxic T lymphocytes (CTLs) from recognizing infected cells. Additionally, IL-18, an IFN-γ-inducing factor, modulates type 1 T helper (Th1) and type 2 T helper (Th2) cell responses while stimulating NK cells and CTLs, which MPXV manipulates to evade detection [4].

### Impaired T-cell response

The regulation of T-cell responses could explain how orthopoxvirus specific memory exists. Without antibodies that neutralize, T cells from immunized primates that are not humans were incapable to protect against deadly MPXV infection. This suggests that compromised T-cell responses, potentially influenced by viral immune evasion strategies, play a crucial role in disease outcomes [35]. By uncovering MPXV's evasion tactics and their effects on immune responses, researchers can develop more effective strategies to combat this virus.

## Mpox immunotherapies

MPXV pathology is related with the seriousness of Mpox disease, and vaccination has shown promise in reducing viral infections and autoimmune disorders. One method to treatment could be focusing on the specific immunological aspects of Mpox.

### Antiviral drugs

Antiviral medicines have an important role in controlling MPXV infection. Antiviral medication should be considered among individuals with severe disease or immune deficiency toddlers beneath the age of 8 years, and pregnant women [44]. Tecovirimat and brincidofovir are two FDA-approved antiviral drugs used to treat smallpox that can also be modified to treat Mpox [45]. The first smallpox medication authorized by the FDA is called tecovirimat (ST-246), and it works specifically against numerous orthopoxviruses, including Mpox [46–48]. Tecovirimat suppresses the development of the viral envelope by selectively targeting the viral protein called VP37, which has undergone extensive conservation across all orthopoxviruses [49]. In 2022, the FDA and EMA granted tecovirimat a license to treat Mpox [47, 50]. Brincidofovir (CMX001), a fat-soluble derivative of cidofovir, has broad antiviral activity targeting double-stranded DNA viruses by inhibiting DNA polymerase [51, 52]. Additionally, eye drops or ointments comprising trifluridine and vidarabine have been used for treating Mpox-related lesions [53, 54]. Recent drug repurposing studies by Akazawa et al. have identified several promising anti-MPXV compounds. Molnupiravir, atovaquone, and mefloquine demonstrated over 50% inhibition, proving more effective than cidofovir. Additionally, they reported that atovaquone and tecovirimat combined therapy increases viral clearance without having any discernible adverse effects [55].

### Intravenous immunoglobulin (IVIG)

Pooled polyclonal immunoglobulin - IVIG was isolated from the plasma of hundreds of healthy donors. IVIG has been used to treat a wide range of immunological, rheumatologic, dermatological, and neurological illnesses. IVIG optimizes the immune response by lowering Kupffer cell activity, decreasing endogenous antibody formation, auto-reactive T cells, and maintaining a balanced cytokine profile [56], Moreover, vaccinia immune globulin (VIG), a recently FDA-approved medication, has shown promise in treating issues that arise after variola virus immunization and could be useful in the fight against MPXV. One important therapeutic option for patients with severe MPXV infections is VIG, when co-administered with antiviral drugs like tecovirimat. [57]. The classification of therapeutic agents and their mechanisms of action are described in Table 1.

**Table 1:** Classification of therapeutic agents and its mechanism of action [22, 44]

Therapeutic agents	Category	Classification	Mechanism of action
Tecovirimat	Antiviral	Orthopoxvirus-specific inhibitor	Inhibits the viral protein VP37, preventing virus maturation and release
Cidofovir		Nucleoside analog	Inhibits viral DNA polymerase, blocking viral replication
Brincidofovir		Nucleoside analog	Similar to cidofovir, it inhibits viral DNA polymerase with reduced toxicity
Vaccinia Immune Globulin	Immunotherapy	Polyvalent immunoglobulin	Contains antibodies that neutralize the virus and provide passive immunity
Imvamune	Vaccine (prevention)	Live attenuated vaccine	Stimulates the immune system to provide protection against orthopoxviruses
ACAM2000		Live attenuated vaccine	Induces immunity by using a replication-competent VACV strain
Jynneos		Modified live attenuated vaccine	Uses a non-replicating Modified Vaccinia Ankara (MVA) virus to stimulate an immune response

### Vaccines

Mpox has resurfaced as a consequence of the withdrawal of the smallpox vaccine in 1980 [38]. In the United States, two vaccines are approved for Mpox preventive measures: ACAM2000 and JYNNEOS. The Board of Advisors on Immunization Practices recommends both immunizations to avoid Mpox. The only vaccine approved for the prevention of Mpox by the FDA and EMA is JYNNEOS/IMVANEX [58, 59]. Table 1 outlines the classification of vaccines and their mechanisms of action. Patients with immune deficiencies are prohibited from receiving ACAM2000 but may get JYNNEOS, IMVAMUNE, or IMVANEX. LC16 m8, an attenuation VACV authorized in Japan since 1975, is alternative third-generation vaccine [60]. These immunizations have a higher safety rating and can be provided to immunocompromised individuals. The effectiveness of these immunizations in Mpox-endemic regions is critical. The smallpox immunization is approximately 85% effective before exposure, but gives less protection after exposure [61]. As a consequence, the vaccine's success depends on how many at-risk populations can be inoculated before sickness [39, 62]. A recent WHO report (August 19, 2024) states that 28 Mpox vaccines are currently in various stages of clinical testing, while 46 vaccines remain in preclinical trials. Ongoing research aims to improve vaccine efficacy and coverage, particularly in endemic region [53].

### Other immunotherapeutic approaches

Immunomodulators, NK cell-based treatments, and monoclonal antibodies (mAbs) are examples of Mpox therapeutics. Recombinant IFNs are one of the immunomodulators that have been authorized to treat hepatitis and SARS-CoV2 viruses. In vitro studies show that IFN suppresses MPXV production and spread, making it a potential therapeutic for Mpox. mAbs have been proposed as treatments for orthopoxvirus infections, although NK cell-based antibody-dependent cytotoxicity is limited to the majority of virus-infected cells. More research is required to understand how NK cell-based therapy works in Mpox [39, 63–67]. Recently, Dutta et al. highlight the immune epitope database, which has reported only eight epitopes from five distinct MPXV proteins [53]. Among them, M1R, A35R, B6R, DNA polymerase, and other proteins have been validated as antigenic candidates, making them potential targets for vaccine development [68]. Additionally, MPXV antigens, such as M1R, E8L, A29L, A35R, and B6R, have shown promise for mRNA and multi-epitope vaccine strategies, capable of eliciting strong immune responses. Beyond vaccines, peptide-based therapeutics, and antibody-based therapy are being developed using MPXV antigens. Key proteins, including H3, A29, A35, E8, B6, and M1, have been identified for vaccine formulation. A mAbs cocktail (c7D11 and c8A) has demonstrated effective inhibition of MPXV, while A29L-derived mAbs aid in rapid viral detection. Structural proteins such as p37 and topoisomerase 1 serve as drug targets, while L6R and A35R play essential roles in vaccine and diagnostic development [53].

## Circadian rhythm and its impact on vaccine efficacy, immunity, and immune response

The innate and adaptive immunity are influenced by the circadian rhythm, which is essential for controlling immunological responses [1]. The proliferation of immune-related genes and pathways, including cytokine synthesis, the presentation of antigens, and PRR activity, is coordinated by circadian components like circadian locomotor output cycles kaput (CLOCK), brain and muscle ARNT-like 1 (BMAL1), period (PER), and cryptochrome (CRY). These treatments improve the immune system's capacity to fight viral infections and assist it in adjusting to changes in the everyday environment. As seen in diseases such as SARS-CoV-2, influenza, and hepatitis C virus (HCV), disruption of this rhythm lessens the effectiveness of immune responses and increases vulnerability to viral replication. It also influences immunological activation and antibody generation, which impacts vaccine efficacy. Studies have indicated that vaccinations given at specific periods of the day have superior results. For example, compared to evening dosages, morning vaccines for strains of influenza A and B have demonstrated better immunogenicity. The significance of coordinating vaccination schedules via circadian cycles to improve vaccine efficacy is highlighted by these time-dependent effects. Furthermore, circadian rhythms affect drug pharmacokinetics, which includes metabolism and bioavailability, indicating that timing drug delivery can maximize therapeutic results. Comprehending the impact of circadian rhythms on immune response is essential when dealing with newly developing illnesses such as Mpox. The circadian rhythm also affects vital physiological processes that mediate the host's defense against viral infections, including cytokine signaling and macrophage activation. Using this information to create vaccinations and chrono-modulated antiviral treatments could enhance public health responses and disease control [69].

Overall, there can be major advantages in the fight against viral infections from incorporating circadian science into immunological investigation and therapeutic procedures. Healthcare practices can improve vaccine-induced protection and reduce infection severity by aligning vaccination schedules, medication delivery, and therapeutic interventions with the body's circadian clock.

## Future perspectives

Future research on Mpox should focus on several critical areas to deepen understanding and improve treatment strategies. Investigating therapeutics that targets the immunopathology of Mpox infection is crucial, including vaccines and antiviral agents designed to either inhibit viral entry or directly combat the virus. A combined approach, integrating immunotherapies and antiviral drugs, may offer enhanced effectiveness compared to monotherapy. Additionally, the current global Mpox outbreak has disproportionately impacted HIV-positive males, highlighting an urgent need to address this population’s unique vulnerabilities. Limited data exist on the effectiveness of antiviral treatments, though case reports have documented their use in Mpox patient care, underscoring the value and necessity of rigorous clinical investigations. Another challenge is the potential hindrance to antibody-based therapies and next-generation vaccines posed by antibody-dependent enhancement found in numerous viral infections [70, 71]. Furthermore, establishing a standardized, effective treatment protocol, along with identifying prognostic markers for severe cases, is vital to mitigate the risk of a global Mpox resurgence [72]. Addressing these issues is likely to advance knowledge of Mpox immunopathology and immunotherapy, providing a foundation for improved clinical outcomes in severe cases.

## Conclusion

A worldwide health crisis has been proclaimed in response to the recent development of Mpox triggered by MPXV, which has the potential to transmit between species. The virus is divided into two clades: Central African (also known as Congo Basin or Clade I) and West African (Clade II). The Central African Clade (clade 1) is particularly aggressive. Clade II b has been responsible for the present outbreak, which includes atypical clinical evidence, a substantial rise in cases globally, and an abnormal number of diseases in MSM patients. Mpox symptoms comprise fever, lymphadenopathy, breakouts, and tumors, and the virus can transmit through a variety of routes, including animal-to-human, human-to-human, and vertical transmission during pregnancy. Pathophysiology refers to the virus's entry, replication, dispersion, and host reactions. Mpox triggers an immunological response that involves innate immune system cells, B cells, and T cells. Innate immune cells perform two functions: they are the first line of protection against viral illnesses and they might be targets for certain viruses. Determining the role of the innate immune system in MPXV-infected people is critical to discovering biomarkers and determining illness prognosis. B cells and immunoglobulins are crucial in combating poxviruses, with memory B cells and sustained antibody levels showing resilience and longevity, while CD4 + T cells promote memory B-cell development. Genes are used by orthopoxviruses to alter host cell signaling pathways, inhibit virus replication, and regulate apoptosis. They also employ proteins to inhibit cellular activation and antagonize immunological mediators and the existence of Orthopoxvirus-specific memory may be explained by this modification of T-cell response. Without neutralizing antibodies, T cells cannot protect against deadly infections. Understanding these evasion techniques can aid in the development of more effective methods against MPXV. Optimizing vaccine timing and treatments based on circadian rhythms can boost immune responses and improve outcomes in viral infections. Further study is required to comprehend human immunity to Mpox and to develop effective approaches for diagnosis, treatment, and prevention.

## Data Availability

No datasets were generated or analysed during the current study.

## Abbreviations

MPXV: Monkeypox virus
Mpox: Monkeypox
VACV: Vaccinia virus
DRC: Democratic Republic of Congo
WHO: World Health Organization
MSM: Men who have sex with men
EVV: Extracellular enveloped virus
IMV: Intracellular mature virus
NK: Natural killer
TNF: Tumor necrosis factor
IFN-γ: Interferon-gamma
VIG: Vaccinia immune globulin
IFNs: Interferons
MOPICE: Mpox inhibitors of complement enzymes
MHC: Major histocompatibility complex
NF-B: Nuclear factor kappa B cells
IRF3: Interferon-regulated factor 3
PRRs: Pattern recognition receptors
STAT: Signal transducer and promoter of transcription
JAK1: Janus Kinase 1
IKKε: IкB Kinase Epsilon
P: Phosphorylation
TBK1: TANK-binding kinase 1
IRF: Interferon regulatory factor
TYK2: Tyrosine kinase 2
IFNAR: Interferon-Alpha/Beta Receptor
MAVS: Mitochondrial antiviral signalling
eIF2α: Eukaryotic Initiation Factor 2
IVIG: Intravenous immunoglobulin
mAbs: Monoclonal antibodies
CLOCK: Circadian locomotor output cycles kaput
BMAL1: Brain and muscle ARNT-like 1
PER: Period
CRY: Cryptochrome
